# Gut Bacteria Promote Phosphine Susceptibility of *Tribolium castaneum* by Aggravating Oxidative Stress and Fitness Costs

**DOI:** 10.3390/insects14100815

**Published:** 2023-10-15

**Authors:** Zhengyan Wang, Shan Zhang, Zhiyuan Liu, Zhenzhen Chang, Haisheng Hu

**Affiliations:** School of Food and Strategic Reserves, Henan University of Technology, Zhengzhou 450001, China; 2022920144@stu.haut.edu.cn (S.Z.); 2022920138@stu.haut.edu.cn (Z.L.); 2021920132@stu.haut.edu.cn (Z.C.); 2020920229@stu.haut.edu.cn (H.H.)

**Keywords:** red flour beetle, fumigant, pesticide resistance, gut bacteria, fitness cost, oxidative stress

## Abstract

**Simple Summary:**

The red flour beetle, *Tribolium castaneum*, has developed widespread pesticide resistance. Knowledge about resistance mechanisms can provide ideas for pesticide resistance management. Since gut microbes can affect host pesticide resistance, we explored gut bacteria-mediated phosphine susceptibility in *T*. *castaneum* and its molecular basis. Among five cultivable gut bacteria excised from a phosphine-resistant *T*. *castaneum*, only *Enterococcus* sp. inoculation significantly promoted host susceptibility to phosphine, while inoculation of any other gut bacteria did not. Furthermore, when *T*. *castaneum* was exposed to phosphine, *Enterococcus* sp. inoculation decreased the female fecundity, promoted host oxidative stress, and suppressed the expression and activity of host antioxidant enzymes. In the absence of phosphine, *Enterococcus* sp. inoculation also elicited overactive host immune responses, including the dual oxidase–reactive oxygen species system. These results indicate that *Enterococcus* sp. likely promotes host phosphine susceptibility by aggravating oxidative stress and fitness costs.

**Abstract:**

Knowledge about resistance mechanisms can provide ideas for pesticide resistance management. Although several studies have unveiled the positive or negative impacts of gut microbes on host pesticide resistance, minimal research is available regarding the association between gut microbes and host phosphine resistance. To explore the influence of gut bacteria on host phosphine susceptibility and its molecular basis, mortality, fitness, redox responses, and immune responses of adult *Tribolium castaneum* were determined when it was challenged by phosphine exposure and/or gut bacteria inoculation. Five cultivable gut bacteria were excised from a population of phosphine-resistant *T*. *castaneum*. Among them, only *Enterococcus* sp. inoculation significantly promoted host susceptibility to phosphine, while inoculation of any other gut bacteria had no significant effect on host phosphine susceptibility. Furthermore, when *T*. *castaneum* was exposed to phosphine, *Enterococcus* sp. inoculation decreased the female fecundity, promoted host oxidative stress, and suppressed the expression and activity of host superoxide dismutase, catalase, and peroxidase. In the absence of phosphine, *Enterococcus* sp. inoculation also elicited overactive immune responses in *T*. *castaneum*, including the immune deficiency and Toll signaling pathways and the dual oxidase–reactive oxygen species system. These results indicate that *Enterococcus* sp. likely promotes host phosphine susceptibility by aggravating oxidative stress and fitness costs.

## 1. Introduction

The red flour beetle, *Tribolium castaneum* (Herbst, 1797) (Coleoptera: Tenebrionidae), is one of the most damaging pests, which can infest 246 grain commodities and cause economic losses in warehouses and mills worldwide [1]. Product losses posed by *T*. *castaneum* infestations are up to 34–40% [2]. Owing to the magnitude of such losses, it has been controlled with various pesticides over a long period. However, *T*. *castaneum* can easily accommodate to frequently used pesticides, including contact pesticides and the fumigant phosphine. It has developed diverse strategies, including metabolic resistance, increased efflux capacities, and target site insensitivity, to deal with pesticides [3]. Therefore, knowledge of resistance mechanisms is crucial for the development of novel pest management strategies [4]. Recently, the association between gut microbes and host resistance to pesticides and its possible mechanisms have been unveiled in many studies [5], which provides a direction for investigating resistance mechanisms.

Phosphine can suppress the activity of electron transfer chain enzymes and antioxidant enzymes such as catalase and peroxidase, thus eliciting massive accumulation of highly reactive oxygen species (ROS), which contributes to phosphine toxicity [3]. Additionally, considering that infection with a high load of gut bacteria commonly elicits overactive immune responses, subsequently promoting host oxidative stress via a sudden boost of humoral immune effectors such as ROS [6,7], it is assumed that some bacteria can modify host phosphine susceptibility by intervening in the redox system. Furthermore, tradeoffs exist between physiological traits due to competition for limiting resources, i.e., increases in the fitness value of one trait may lead to a corresponding decline in the fitness value of another [8]. Thus, it is speculated that when pests are simultaneously challenged by pesticide exposure and bacterial infection, the tradeoff between immune responses and other fitness traits associated with pesticide resistance will increase host susceptibility to pesticides, as has been found in *Diaphorina citri* infested with *Candidatus* Liberibacter asiaticus [9].

Although several studies have unveiled the positive or negative impacts of gut microbes on host pesticide resistance [5,10], minimal research is available regarding the association between gut microbes and host phosphine resistance [11,12]. Herein, we aim to explore gut bacteria-mediated phosphine susceptibility in *T. castaneum* and its molecular basis, with emphasis on host oxidative stress and fitness costs, through a four-stage research process. When *T. castaneum* was challenged by phosphine exposure and/or gut bacteria inoculation, (1) the mortality of *T. castaneum* was measured to unveil the impact of gut bacteria on host phosphine resistance and to screen resistance-mediating gut bacteria, (2) the female fecundity of *T*. *castaneum* was measured to unveil the impact of resistance-mediating gut bacteria on host reproductive fitness, (3) the level of hydrogen peroxide and malondialdehyde in *T. castaneum* was measured to unveil the impact of resistance-mediating gut bacteria on host oxidative stress, and (4) antioxidant and immune responses in *T. castaneum* were measured to unveil the molecular basis for gut bacteria-induced oxidative stress.

## 2. Materials and Methods

### 2.1. Insects

The stock of *T. castaneum* originated from a population sampled from Shantou, Guangdong, China, in June 2015 and was cultured continuously in laboratory conditions for more than seven years. This stock was strongly resistant to phosphine with a resistance ratio of 208.3, determined via the FAO standard method [13]. The normal diet for the insects was a blend of wholemeal flour and yeast powder (19:1, w:w) sterilized via exposure to ultraviolet radiation (about 254 nm) for 2 h. Insects were cultured at 28 °C and 65% relative humidity in continuous darkness. The pupae were collected and then separated by sex as previously described for subsequent assays [2]. If there were no special instructions, experiments with *T*. *castaneum* were performed under the same normal conditions mentioned above.

### 2.2. Isolation and Identification of Gut Bacteria

To isolate gut bacteria, 7-day-old mixed-sex adult *T. castaneum* were starved for 1 d, then surface disinfected with 70% alcohol, and subsequently rinsed thrice with aseptic water. Gut bacteria were excised and purified as previously described [14]. The purified gut bacteria were collected and preserved in Luria-Bertani (LB) broth added with 25% glycerine at −80 °C for subsequent assays. Bacterial isolates were revived in LB broth prior to use. Five cultivable gut bacteria were successfully excised from the tested *T. castaneum*. Bacterial identification based on 16S rDNA genes was conducted as previously described [15]. The 16S rDNA gene primers 27F and 1492R were used to amplify DNA fragments (Supplementary Table S1). Amplified DNA fragments were sequenced and blasted against the 16S rDNA database and submitted to the NCBI database, and their GenBank accession numbers were OM992224.1, OM992227.1, OM992228.1, OM992229.1, and OM992230.1 (Supplementary Figure S1).

### 2.3. Antibiotic Treatment and Gnotobiotic Inoculation

Since antibiotics could reshape and deplete insect gut bacteria [16], newly emerged beetles were cultured with a normal diet containing 20 μg/g antibiotic cocktail (ciprofloxacin:penicillin:vancomycin = 1:1:1) for 3 d to produce germ-free beetles. After antibiotic treatment, 4-day-old germ-free beetles were cultured with a normal diet mixed with a given bacterial isolate for 3 d to produce gnotobiotic beetles [15]. All treatments were conducted in an aseptic environment. Germ-free and gnotobiotic beetles were then cultured with a normal diet in an aseptic environment for subsequent assays. The beetles cultured with a normal diet under normal conditions were used as conventional beetles.

To confirm whether gut bacteria were successfully depleted and inoculated, their abundance was determined via real-time quantitative PCR (qRT-PCR) using the 2^−ΔΔCT^ method [17] and normalized by the qRT-PCR data of the *β-actin* gene of *T. castaneum*. Gut homogenates of thirty 6-, 8-, 10-, 12-, 14-, and 16-day-old beetles were prepared according to the method described above. Total DNA was extracted from the gut homogenate using an E.Z.N.A.^®^ soil DNA kit (Omega Bio-Tek, Norcross, GA, USA). qRT-PCR primers are shown in Supplementary Table S1. The qRT-PCR was run in a 20 μL reactive mixture including 10 μL of SYBR Green Mix (TaKaRa, Otsu, Japan), 0.8 μL of each primer (50 μmol/L), and 5 ng of template DNA. Thermocycling conditions were 95 °C for 30 s, and then 35 cycles of 95 °C for 5 s, 60 °C for 30 s, and 72 °C for 30 s. After the thermal cycles, melting curve analysis was conducted to confirm that the reaction produced a single, specific product. The thermocycling programs for the melting curve analysis were the same as those previously described [6]. Reactions were conducted in triplicate, and the mean of triplicate analyses represented one biological repetition. Each treatment consisted of five biological repetitions.

### 2.4. Bioassay of Phosphine Susceptibility

Phosphine susceptibility of conventional, germ-free, and gnotobiotic beetles was measured according to the FAO standard method [13] to unveil the impact of bacteria inoculation on host phosphine resistance. Thirty 10-day-old beetles were exposed to phosphine at the concentrations of 0, 1000, 1250, 1500, 1750, and 2000 mL/m^3^ for 20 h. After exposure to phosphine, the beetles were ventilated and then cultured under normal conditions. Their mortality was assessed after 14 d of culture and corrected for control mortality [18]. Each treatment consisted of five biological repetitions. Since only *Enterococcus* sp. significantly influenced host phosphine susceptibility, it was used in subsequent assays to explore the molecular basis for gut bacteria-mediated phosphine susceptibility in *T*. *castaneum*.

### 2.5. Determination of Female Fecundity

Female fecundity of conventional, germ-free, and gnotobiotic beetles was measured to assess the impact of *Enterococcus* sp. inoculation on reproductive fitness of *T*. *castaneum* after exposure to phosphine. After exposure of 20 pairs of 10-day-old beetles to 1250 mL/m^3^ phosphine for 20 h, the beetles were ventilated and then cultured under normal conditions. Pairs were separately cultured with a normal diet in a culture plate with a diameter of 15 mm and checked daily for 45 d, on which most females did not lay eggs anymore. The diet was changed every 2 d. Freshly laid eggs were transferred to a new culture plate and monitored for 10 d for emergence. Female fecundity was defined as the number of viable eggs laid per female. The average fecundity of 20 pairs represented one biological repetition. Each treatment consisted of five biological repetitions.

### 2.6. Qualification of MDA and H_2_O_2_

Since malondialdehyde (MDA) and hydrogen peroxide (H_2_O_2_) are commonly used as biological indicators of oxidative stress [19,20], the level of MDA and H_2_O_2_ in conventional, germ-free, and gnotobiotic beetles was measured to assess the impact of *Enterococcus* sp. inoculation on oxidative stress in *T. castaneum* after exposure to phosphine. After exposure of 10-day-old beetles to 1250 mL/m^3^ phosphine for 0, 6, 12, and 24 h, the level of MDA and H_2_O_2_ was determined using BC0020 and BC3590 assay kits (Solarbio, Beijing, China), respectively. MDA reacts with thiobarbituric acid to produce tridione with a maximum absorbance at 532 nm, and H_2_O_2_ reacts with titanic sulfate to produce a titanium peroxide complex with characteristic absorbance at 415 nm [21]. The level of MDA and H_2_O_2_ was calculated based on the absorbance according to the assay kit protocol. The samples were conducted in triplicate, and the mean of triplicate analyses represented one biological repetition. Each treatment consisted of five biological repetitions.

### 2.7. Determination of SOD, CAT, and POD Activities

The activity of three antioxidant enzymes, namely, superoxide dismutase (SOD), catalase (CAT), and peroxidase (POD), in conventional, germ-free, and gnotobiotic beetles was measured to assess the impact of *Enterococcus* sp. inoculation on the antioxidant system of *T. castaneum* after exposure to phosphine. After exposure of 10-day-old beetles to 1250 mL/m^3^ phosphine for 0, 6, 12, and 24 h, the activity of SOD, CAT, and POD was determined using BC0170, BC0200, and BC0090 assay kits (Solarbio) according to the protocols of the manufacturer, respectively.

SOD activity was determined by detecting superoxide radicals generated by xanthine oxidase and hypoxanthine and monitoring absorbance at 560 nm [22]. One unit of SOD activity refers to the quantity of enzyme necessary to display 50% dismutation of the superoxide radical. CAT activity was determined by detecting the decomposition rate of H_2_O_2_ and monitoring absorbance at 240 nm [23]. One unit of CAT activity refers to the quantity of CAT necessary to decompose 1 µmol of H_2_O_2_ per min per g of sample. POD activity was determined by detecting guaiacol oxidation and monitoring absorbance at 470 nm [24]. One unit of POD activity refers to a 0.01 change in absorbance at 470 nm per min per g of sample per mL of the reactive mixture. The samples were conducted in triplicate, and the mean of triplicate analyses represented one biological repetition. Each treatment consisted of five biological repetitions.

### 2.8. Determination of SOD, CAT, and POD Gene Expression

The expression levels of SOD, CAT, and POD genes in conventional, germ-free, and gnotobiotic beetles were measured to explore the molecular basis for the impact of *Enterococcus* sp. inoculation on the antioxidant system in *T. castaneum* after exposure to phosphine. After exposure of 10-day-old beetles to 1250 mL/m^3^ phosphine for 0, 6, 12, and 24 h, the total RNA of 50 beetles was extracted using TRIzol^®^ reagent (Accurate Biotechnology, Changsha, China). Contaminating genomic DNA was depleted using DNase I (Accurate Biotechnology). Subsequently, cDNA libraries were constructed from 1 μg of RNA using M-MLV Reverse Transcriptase (Accurate Biotechnology). The relative gene expression data were assessed using the 2^−ΔΔCT^ method and normalized with the qRT-PCR data of the *Rps18* gene of *T. castaneum* [25]. qRT-PCR primers are shown in Supplementary Table S1. The 20 μL qRT-PCR reactive mixture included 10 μL of SYBR Green Mix (TaKaRa, Otsu, Japan), 0.8 μL of each primer (50 μmol/L), and 2 μL of cDNA (diluted 1:10). qRT-PCR conditions were the same as those described above. Reactions were conducted in triplicate, and the mean of triplicate analyses represented one biological repetition. Each treatment consisted of five biological repetitions.

### 2.9. Measurement of Immune Responses

The expression levels of *PLCβ*, *Atf2*, and *DUOX* from the dual oxidase-reactive oxygen species (DUOX-ROS) system, *IMD* from the immune deficiency (IMD) signaling pathway, and *Toll* from the Toll signaling pathway in conventional, germ-free, and gnotobiotic beetles were measured to assess the impact of *Enterococcus* sp. inoculation on host immune responses in the absence of phosphine. The total RNA of fifty 6-, 8-, 10-, 12-, 14-, and 16-day-old beetles was sequentially extracted, processed, and analyzed according to the method described above. qRT-PCR primers are shown in Supplementary Table S1. Reactions were conducted in triplicate, and the mean of triplicate analyses represented one biological repetition. Each treatment consisted of five biological repetitions.

### 2.10. Statistical Analysis

Datasets were tested for normality using the Kolmogorov–Smirnov test. All datasets were normally distributed and statistically compared with one-way analysis of variance (ANOVA) and Tukey’s honest significant difference (HSD) test. All statistics were conducted using SPSS Statistics 22.0 (IBM, NY, USA), and differences among means were considered significant at *p* < 0.05.

## 3. Results

### 3.1. Impact of Bacterial Treatment on Bacterial Abundance

**Figure 1:**
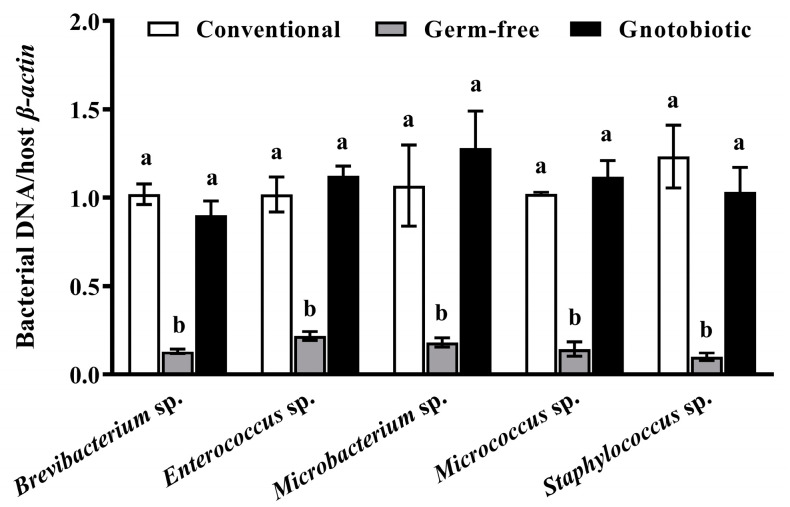
Mean ± SE relative gut load of *Brevibacterium* sp., *Enterococcus* sp., *Microbacterium* sp., *Micrococcus* sp., and *Staphylococcus* sp. in adult *Tribolium castaneum* with different treatments of gut bacteria (*n* = 5). Germ-free means that gut bacteria are successfully depleted from beetles via antibiotic treatment. Gnotobiotic means that a given gut bacterium is successfully inoculated into germ-free beetles. The same letters indicate no significant difference among the means in the same cluster (one-way ANOVA and Tukey HSD test, *p* > 0.05).

Five cultivable bacteria, namely, *Brevibacterium* sp., *Enterococcus* sp., *Microbacterium* sp., *Micrococcus* sp., and *Staphylococcus* sp., were excised from the gut of phosphine-resistant *T. castaneum* (Supplementary Figure S1). Since germ-free and gnotobiotic beetles were used to assess the impact of *Enterococcus* sp. on host traits, the methodology for producing these beetles required technical approval. The gut load of *Brevibacterium* sp., *Enterococcus* sp., *Microbacterium* sp., *Micrococcus* sp., and *Staphylococcus* sp. (Figure 1) varied substantially among conventional, germ-free, and gnotobiotic beetles (*F*_2,12_ = 68.40, *p* < 0.001; *F*_2,12_ = 55.57, *p* < 0.001; *F*_2,12_ = 10.60, *p* = 0.002; *F*_2,12_ = 84.91, *p* < 0.001; *F*_2,12_ = 21.46, *p* < 0.001, respectively). Five gut bacteria were successfully depleted via antibiotic treatment to produce germ-free beetles, and the gut load of a given bacteria was restored to its normal level after inoculation of germ-free beetles with the bacterial isolate, thus producing gnotobiotic beetles.

### 3.2. Impact of Bacteria Inoculation on Host Phosphine Susceptibility

**Figure 2:**
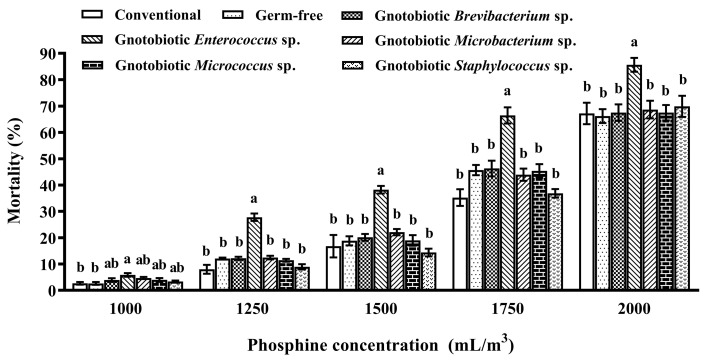
Mean ± SE mortality of adult *Tribolium castaneum* after exposure to different concentrations of phosphine for 20 h (*n* = 5). Germ-free means that gut bacteria are successfully depleted from beetles via antibiotic treatment. Gnotobiotic means that a given gut bacterium is successfully inoculated into germ-free beetles. The same letters indicate no significant difference among the means in the same cluster (one-way ANOVA and Tukey HSD test, *p* > 0.05).

The impact of five gut bacterial isolates on host phosphine susceptibility was assessed to screen resistance-mediating bacteria. *Enterococcus* sp. inoculation significantly promoted host susceptibility to phosphine, and the mortality of the beetles inoculated with *Enterococcus* sp. was substantially higher than those of conventional and germ-free beetles after exposure to 1000, 1250, 1500, 1750, and 2000 mL/m^3^ phosphine for 20 h (*F*_6,28_ = 4.09, *p* = 0.005; *F*_6,28_ = 42.72, *p* < 0.001; *F*_6,28_ = 13.21, *p* < 0.001; *F*_6,28_ = 15.54, *p* < 0.001; *F*_6,28_ = 4.26, *p* = 0.004, respectively) (Figure 2). Moreover, inoculation of any other gut bacterial isolate had no significant effect on host phosphine susceptibility, and no significant difference was found in the mortality between germ-free and gnotobiotic beetles. Therefore, *Enterococcus* sp. was used in subsequent assays to explore molecular mechanisms of gut bacteria-induced phosphine susceptibility in *T*. *castaneum*.

### 3.3. Impact of Enterococcus sp. Inoculation on Host Fecundity

**Figure 3:**
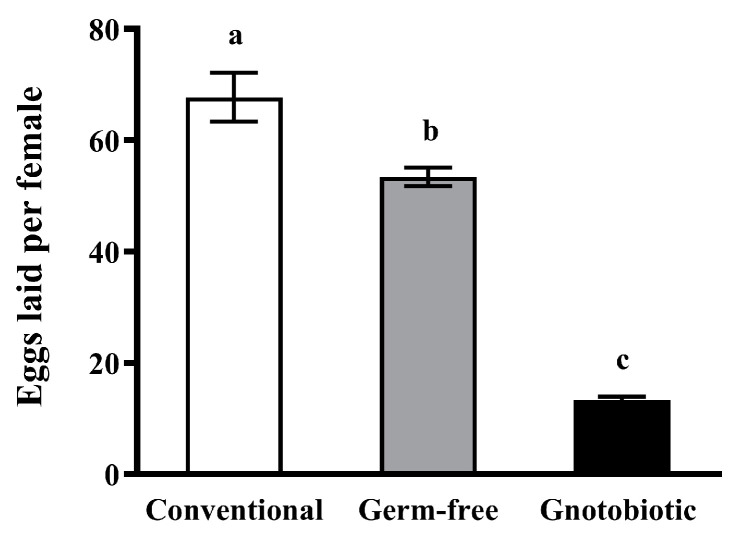
Mean ± SE number of eggs laid per female after exposure of adult *Tribolium castaneum* to 1250 mL/m^3^ phosphine for 20 h (*n* = 5). Germ-free means that *Enterococcus* sp. is successfully depleted from beetles via antibiotic treatment. Gnotobiotic means that *Enterococcus* sp. is successfully inoculated into germ-free beetles. The same letters indicate no significant difference among different treatments (one-way ANOVA and Tukey HSD test, *p* > 0.05).

*Enterococcus* sp. inoculation significantly reduced the female fecundity of *T. castaneum*. After exposure of beetles to 1250 mL/m^3^ phosphine for 20 h, the number of eggs laid per female of gnotobiotic beetles was substantially lower than that of conventional and germ-free beetles (*F*_2,57_ = 106.20, *p* < 0.001) (Figure 3), suggesting that *Enterococcus* sp. inoculation results in fitness costs in *T. castaneum* after exposure to phosphine.

### 3.4. Impact of Enterococcus sp. Inoculation on Host Oxidative Stress

**Figure 4:**
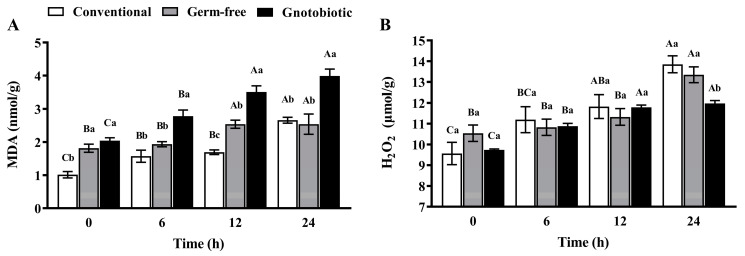
Mean ± SE level of malondialdehyde (MDA, **A**) and hydrogen peroxide (H_2_O_2_, **B**) in adult *Tribolium castaneum* after exposure to 1250 mL/m^3^ phosphine for different periods (*n* = 5). Germ-free means that *Enterococcus* sp. is successfully depleted from beetles via antibiotic treatment. Gnotobiotic means that *Enterococcus* sp. is successfully inoculated into germ-free beetles. The same uppercase letters indicate no significant difference among the means with the same treatment of gut bacteria across different exposure periods, and the same lowercase letters indicate no significant difference among the means of conventional, germ-free, and gnotobiotic beetles within the same exposure period (one-way ANOVA and Tukey HSD test, *p* > 0.05).

After exposure to 1250 mL/m^3^ phosphine for 0, 6, 12, and 24 h, the level of MDA and H_2_O_2_ in *T. castaneum* was measured to assess the impact of phosphine exposure and *Enterococcus* sp. inoculation on host oxidative stress. Phosphine exposure induced serious oxidative stress in *T. castaneum*, and the MDA level (Figure 4A) in conventional, germ-free, and gnotobiotic beetles increased as the phosphine exposure period was extended (*F*_3,16_ = 33.78, *p* < 0.001; *F*_3,16_ = 4.64, *p* = 0.016; *F*_3,16_ = 24.30, *p* < 0.001, respectively). *Enterococcus* sp. inoculation further promoted oxidative stress, and the MDA level in gnotobiotic beetles was substantially higher than that in germ-free beetles after exposure to phosphine for 6, 12, and 24 h (*F*_2,12_ = 15.67, *p* < 0.001; *F*_2,12_ = 45.98, *p* < 0.001; *F*_2,12_ = 13.44, *p* = 0.001, respectively).

Phosphine-induced oxidative stress in *T. castaneum* was further verified by massive accumulation of H_2_O_2_. The H_2_O_2_ level in conventional and germ-free beetles (Figure 4B) increased as the phosphine exposure period was extended (*F*_3,16_ = 10.64, *p* < 0.001; *F*_3,16_ = 10.41, *p* < 0.001, respectively). However, *Enterococcus* sp. inoculation inhibited H_2_O_2_ production in *T. castaneum* after exposure to phosphine, and the H_2_O_2_ level in gnotobiotic beetles was substantially lower than that in germ-free beetles after exposure to phosphine for 24 h (*F*_2,12_ = 8.60, *p* = 0.005).

### 3.5. Impact of Enterococcus sp. Inoculation on the Host Antioxidant System

**Figure 5:**
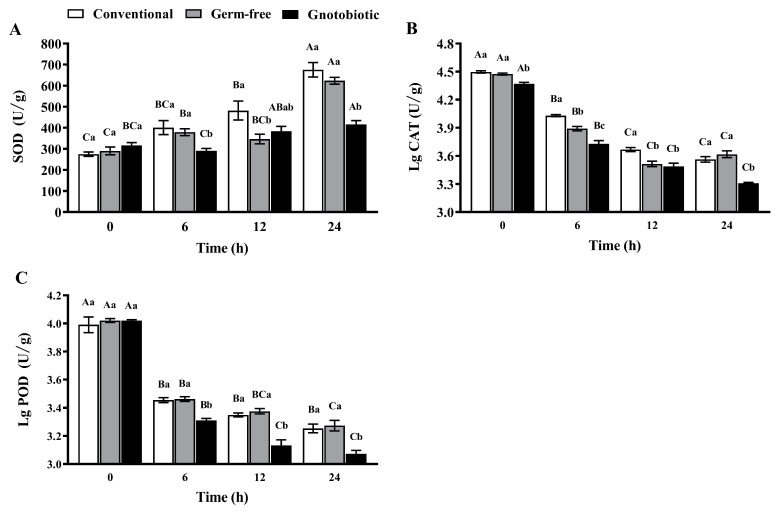
Mean ± SE activity of superoxide dismutase (SOD, **A**), catalase (CAT, **B**), and peroxidase (POD, **C**) in adult *Tribolium castaneum* after exposure to 1250 mL/m^3^ phosphine for different periods (*n* = 5). Germ-free means that *Enterococcus* sp. is successfully depleted from beetles via antibiotic treatment. Gnotobiotic means that *Enterococcus* sp. is successfully inoculated into germ-free beetles. The same uppercase letters indicate no significant difference among the means with the same treatment of gut bacteria across different exposure periods, and the same lowercase letters indicate no significant difference among the means of conventional, germ-free, and gnotobiotic beetles within the same exposure period (one-way ANOVA and Tukey HSD test, *p* > 0.05).

The impact of *Enterococcus* sp. inoculation on the activity of SOD, CAT, and POD in *T. castaneum* was assessed after exposure to 1250 mL/m^3^ phosphine for 0, 6, 12, and 24 h. Phosphine exposure promoted SOD activity in conventional, germ-free, and gnotobiotic beetles, which increased as the phosphine exposure period was extended (*F*_3,16_ = 25.56, *p* < 0.001; *F*_3,16_ = 61.87, *p* < 0.001; *F*_3,16_ = 11.28, *p* < 0.001, respectively) (Figure 5A). However, *Enterococcus* sp. inoculation suppressed SOD activity in *T*. *castaneum* after exposure to phosphine. SOD activity in gnotobiotic beetles was substantially lower than that in germ-free beetles after exposure to phosphine for 6 and 24 h (*F*_2,12_ = 6.80, *p* = 0.011; *F*_2,12_ = 31.60, *p* < 0.001, respectively).

However, phosphine exposure suppressed the activity of CAT (Figure 5B) and POD (Figure 5C), which declined in conventional, germ-free, and gnotobiotic beetles as the phosphine exposure period was extended (*F*_3,16_ = 748.39, *p* < 0.001; *F*_3,16_ = 768.52, *p* < 0.001; *F*_3,16_ = 424.97, *p* < 0.001 for CAT, respectively; *F*_3,16_ = 33.38, *p* < 0.001; *F*_3,16_ = 431.92, *p* < 0.001; *F*_3,16_ = 1474.92, *p* < 0.001 for POD, respectively). *Enterococcus* sp. inoculation further suppressed the activity of CAT and POD. CAT activity in gnotobiotic beetles was substantially lower than that in germ-free beetles after exposure to phosphine for 6 and 24 h (*F*_2,12_ = 46.52, *p* < 0.001; *F*_2,12_ = 19.91, *p* < 0.001, respectively), and POD activity in gnotobiotic beetles was substantially lower than that in germ-free beetles after exposure to phosphine for 6, 12, and 24 h (*F*_2,12_ = 24.26, *p* < 0.001; *F*_2,12_ = 27.93, *p* < 0.001; *F*_2,12_ = 9.83, *p* = 0.003, respectively).

**Figure 6:**
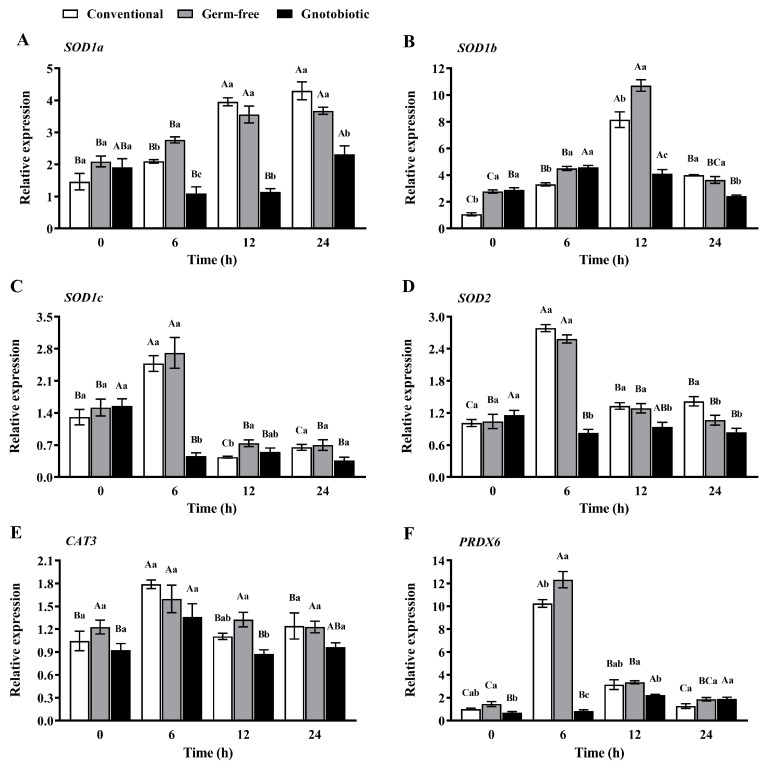
Mean ± SE relative expression levels of *SOD1a* (**A**), *SOD1b* (**B**), *SOD1c* (**C**), *SOD2* (**D**), *CAT3* (**E**), and *PRDX6* (**F**) in adult *Tribolium castaneum* after exposure to 1250 mL/m^3^ phosphine for different periods (*n* = 5). Germ-free means that *Enterococcus* sp. is successfully depleted from beetles via antibiotic treatment. Gnotobiotic means that *Enterococcus* sp. is successfully inoculated into germ-free beetles. The same uppercase letters indicate no significant difference among the means with the same treatment of gut bacteria across different exposure periods, and the same lowercase letters indicate no significant difference among the means of conventional, germ-free, and gnotobiotic beetles within the same exposure period (one-way ANOVA and Tukey HSD test, *p* > 0.05).

The expression levels of *SOD1a*, *SOD1b*, *SOD1c* (three transcript variants of the *SOD1* gene in *T. castaneum*), *SOD2*, *CAT3*, and *PRDX6* in *T. castaneum* were measured after exposure to 1250 mL/m^3^ phosphine for 0, 6, 12, and 24 h (Figure 6). Phosphine exposure led to upregulation of SOD gene expression. The expression levels of *SOD1a*, *SOD1b*, *SOD1c*, and *SOD2* first increased and then decreased in conventional, germ-free, and gnotobiotic beetles as the phosphine exposure period was extended (*F*_3,16_ = 47.88, *p* < 0.001; *F*_3,16_ = 18.37, *p* < 0.001; *F*_3,16_ = 7.12, *p* = 0.003 for *SOD1a*, respectively; *F*_3,16_ = 94.45, *p* < 0.001; *F*_3,16_ = 179.90, *p* < 0.001; *F*_3,16_ = 29.06, *p* < 0.001 for *SOD1b*, respectively; *F*_3,16_ = 54.07, *p* < 0.001; *F*_3,16_ = 21.35, *p* < 0.001; *F*_3,16_ = 29.63, *p* < 0.001 for *SOD1c*, respectively; *F*_3,16_ = 123.62, *p* < 0.001; *F*_3,16_ = 54.13, *p* < 0.001; *F*_3,16_ = 3.92, *p* = 0.028 for *SOD2*, respectively), reaching the maximal values after exposure to phosphine for 24, 12, 6, and 6 h, respectively.

However, *Enterococcus* sp. inoculation led to downregulation of SOD gene expression in *T. castaneum* after exposure to phosphine. The expression level of *SOD1a* in gnotobiotic beetles was substantially lower than that in germ-free beetles after exposure to phosphine for 6, 12, and 24 h (*F*_2,12_ = 39.10, *p* < 0.001; *F*_2,12_ = 72.94, *p* < 0.001; *F*_2,12_ *=* 19.18, *p* < 0.001, respectively). Similarly, the expression level of *SOD1b* in gnotobiotic beetles was substantially lower than that in germ-free beetles after exposure to phosphine for 12 and 24 h (*F*_2,12_ = 53.72, *p* < 0.001; *F*_2,12_ = 28.17, *p* < 0.001, respectively), the expression level of *SOD1c* in gnotobiotic beetles was substantially lower than that in germ-free beetles after exposure to phosphine for 6 h (*F*_2,12_ = 31.13, *p* < 0.001), and the expression level of *SOD2* in gnotobiotic beetles was substantially lower than that in germ-free beetles after exposure to phosphine for 6 and 12 h (*F*_2,12_ = 243.76, *p* < 0.001; *F*_2,12_ = 7.41, *p* = 0.008, respectively).

Similar variation trends in CAT and POD gene expression occurred in *T*. *castaneum* when challenged by phosphine exposure and *Enterococcus* sp. inoculation. The expression level of *CAT3* first increased and then decreased in conventional and gnotobiotic beetles as the phosphine exposure period was extended (*F*_3,16_ = 9.12, *p* = 0.001; *F*_3,16_ = 4.60, *p* = 0.017, respectively). The expression level of *PRDX6* first increased and then decreased in conventional and germ-free beetles as the phosphine exposure period was extended (*F*_3,16_ = 219.17, *p* < 0.001; *F*_3,16_ = 174.68, *p* < 0.001, respectively). The expression levels of *CAT3* and *PRDX6* reached the maximal values after exposure to phosphine for 6 h. *Enterococcus* sp. inoculation led to downregulation of the expression of *CAT3* and *PRDX6* in *T. castaneum* after exposure to phosphine. The expression level of *CAT3* in gnotobiotic beetles was substantially lower than that in germ-free beetles after exposure to phosphine for 12 h (*F*_2,12_ = 11.03, *p* < 0.001), and the expression level of *PRDX6* in gnotobiotic beetles was substantially lower than that in germ-free beetles after exposure to phosphine for 0, 6, and 12 h (*F*_2,12_ = 6.91, *p* = 0.010; *F*_2,12_ = 176.51, *p* < 0.001; *F*_2,12_ = 5.29, *p* = 0.023, respectively).

### 3.6. Impact of Enterococcus sp. Inoculation on Host Immune Responses

**Figure 7:**
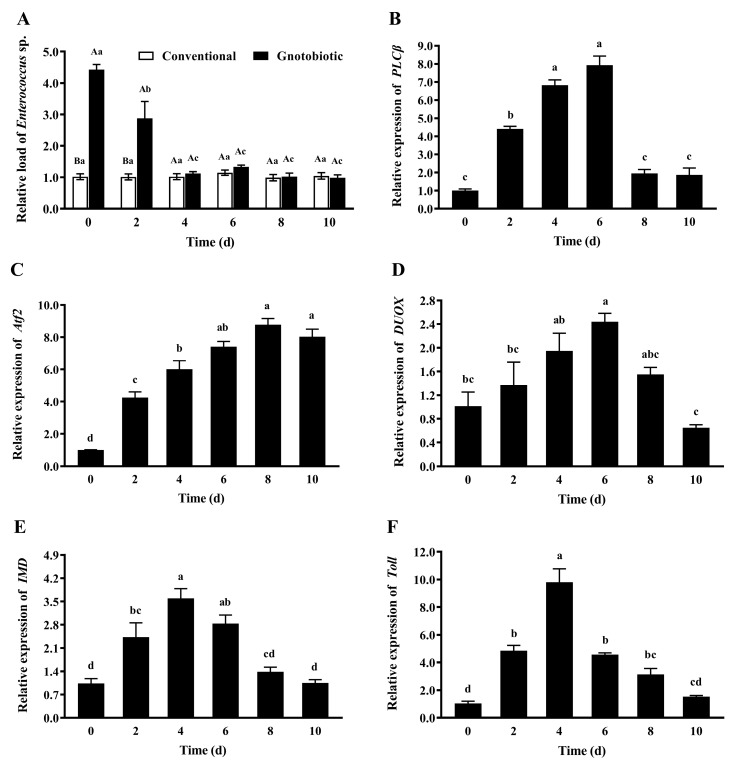
Mean ± SE relative gut load of *Enterococcus* sp. (**A**) and relative expression levels of *PLCβ* (**B**), *Atf2* (**C**), *DUOX* (**D**), *IMD* (**E**), and *Toll* (**F**) in adult *Tribolium castaneum* after inoculation of *Enterococcus* sp. into germ-free beetles for different periods (*n* = 5). Gnotobiotic means that *Enterococcus* sp. is successfully inoculated into germ-free beetles. The same uppercase letters indicate no significant difference among the means of conventional and gnotobiotic beetles within the same period (one-way ANOVA, *p* > 0.05), and the same lowercase letters indicate no significant difference among the means with the same gut bacteria treatment across different periods (one-way ANOVA and Tukey HSD test, *p* > 0.05).

To assess the impact of *Enterococcus* sp. inoculation on host immune responses, the gut load of *Enterococcus* sp. and the expression levels of *PLCβ*, *Atf2*, *DUOX*, *IMD*, and *Toll* in gnotobiotic *T*. *castaneum* were evaluated on days 0, 2, 4, 6, 8, and 10 after inoculation of *Enterococcus* sp. to germ-free beetles. After *Enterococcus* sp. was inoculated into germ-free beetles, the gut load of *Enterococcus* sp. in gnotobiotic beetles (Figure 7A) was significantly higher than that in conventional beetles during the first two days after bacteria inoculation (*F*_1,8_ = 313.73, *p* < 0.001; *F*_1,8_ = 11.77, *p* = 0.009) and recovered to the normal level from the fourth day after bacteria inoculation (*F*_1,8_ = 0.89, *p* = 0.374 for the fourth day; *F*_1,8_ = 3.40, *p* = 0.103 for the sixth day; *F*_1,8_ = 0.06, *p* = 0.808 for the eighth day; *F*_1,8_ = 0.19, *p* = 0.674 for the tenth day).

The expression levels of *PLCβ* (Figure 7B), *Atf2* (Figure 7C), *DUOX* (Figure 7D), *IMD* (Figure 7E), and *Toll* (Figure 7F) first increased and then decreased (*F*_5,24_ = 91.72, *p* < 0.001; *F*_5,24_ = 56.96, *p* < 0.001; *F*_5,24_ = 7.46, *p* < 0.001; *F*_5,24_ = 17.71, *p* < 0.001; *F*_5,24_ = 45.87, *p* < 0.001, respectively), reaching the maximal values on days 6, 8, 6, 4, and 4 after bacteria inoculation, respectively. These results suggest that *T*. *castaneum* recruits different immune signaling pathways to cope with a high gut load of *Enterococcus* sp. and maintain microbial homeostasis.

## 4. Discussion

Five cultivable gut bacteria, namely, *Brevibacterium* sp., *Enterococcus* sp., *Microbacterium* sp., *Micrococcus* sp., and *Staphylococcus* sp., were excised from phosphine-resistant *T. castaneum*. Gut microbes may increase host pesticide resistance by directly degrading pesticides [10], modulating the host immune system [26], and producing nutrients and other beneficial chemicals [27,28]. However, *Enterococcus faecalis* inoculation impaired phosphine resistance of adult *T. castaneum* [12]. In this study, inoculation with another *Enterococcus* sp. also promoted host phosphine susceptibility, while inoculation of any other gut bacteria had no significant effect on host phosphine susceptibility. The negative influence of gut microbes on host pesticide resistance to contact pesticides, which has also been found in other studies [29,30], will provide ideas for pesticide resistance management by integrating pesticides with biocontrol agents containing resistance-mediating bacteria [31,32].

Phosphine exposure elicited an increased level of MDA and H_2_O_2_, indicating high oxidative stress in *T. castaneum*. On the other hand, inoculation with a high load of *Enterococcus* sp. upregulated the expression of *PLCβ*, *Atf2*, and *DUOX* from the DUOX–ROS system in *T. castaneum*, further aggravating oxidative stress in *T. castaneum* [7]. Furthermore, when *T. castaneum* was exposed to phosphine, *Enterococcus* sp. inoculation suppressed the activity of host antioxidant enzymes, including SOD, CAT, and POD, which play a vital role in scavenging ROS [33]. Additionally, since SOD is responsible for the transformation of active superoxide anions into stable H_2_O_2_, a decreased level of H_2_O_2_ formation in *T. castaneum* due to the suppression of SOD activity by *Enterococcus* sp. inoculation will further aggravate oxidative stress. Considering that phosphine-induced oxidative stress and its damage are considered to be among the primary mechanisms of phosphine toxicity [34], it is not difficult to infer that increased mortality rates of *T. castaneum* simultaneously challenged by phosphine exposure and *Enterococcus* sp. inoculation could be attributed to overgeneration of ROS, which will cause damage to essential cellular building blocks, including DNA, proteins, and lipids [35].

Besides the DUOX–ROS system, other immune responses, including IMD and Toll signaling pathways, were also activated by *Enterococcus* sp. inoculation. As an essential life-preserving process, the immune response is privileged over other processes regarding energy supply [36]. The tradeoff between immune responses and other fitness traits associated with pesticide resistance because of alternative allocation of limiting energetic resources will increase host susceptibility to pesticides. For example, infection of *Culex pipiens* with the bacterium *Wolbachia* increased the fitness cost of resistance to pesticides, rendering hosts more susceptible to pesticides [37], and parasitism by *Vavraia culicis* caused fitness costs in *Culex pipiens quinquefasciatus* bearing organophosphate resistance alleles, potentially altering the strength and direction of selection against resistance mutations in untreated environments [38]. Since both antioxidant and immune responses are energetically costly [8,39], it is speculated that tradeoff between them will lead to *Enterococcus* sp.-induced phosphine susceptibility in *T. castaneum*.

During the initial stage of phosphine fumigation, *T*. *castaneum* attempted to mitigate oxidative stress from phosphine by upregulating the expression of *SOD1a, SOD1b, SOD1c, SOD2, CAT3,* and *PRDX6* and promoting SOD activity, as has been found in other phosphine-resistant insects [40]. However, *Enterococcus* sp. inoculation constantly suppressed the expression and activity of SOD, CAT, and POD in *T*. *castaneum* after exposure to phosphine, demonstrating that beetles inoculated with *Enterococcus* sp. could not allocate enough resources to contradict oxidative stress due to tradeoff between immune and antioxidant responses. Similarly, infection of *D*. *citri* with Ca. Liberibacter asiaticus reduced the activity of host detoxifying enzymes such as glutathione-S-transferase (GST) and cytochrome P_450_ [31], and infection with a high titer of Phytoplasma inhibited the activity of host detoxifying enzymes such as β-esterase and GST in *Amplicephalus curtulus* [41]. Meanwhile, *Enterococcus* sp. inoculation decreased reproductive fitness of *T*. *castaneum* after exposure to phosphine, suggesting that beetles could not allocate enough resources to conduct normal physiological activities. Increased mortality, reduced antioxidant enzyme activity, and low fecundity indicate increased fitness costs in *T*. *castaneum* simultaneously challenged by phosphine exposure and *Enterococcus* sp. inoculation. From an evolutionary perspective, gut microbe-induced fitness costs will delay the development of phosphine resistance among insect populations.

Previous research showed that bacteria inoculation can activate the Toll signaling pathway and subsequently suppress the insulin signaling pathway [36]. The low expression of AGC kinase (Akt), an element in the insulin signaling pathway, can inhibit the activity of the downstream transcription factor erythroid 2-related factor 2 (Nrf2). Thus, Nrf2 cannot translocate into the nucleus to activate antioxidant enzyme expression [42]. Therefore, it is speculated that *Enterococcus* sp. regulates the expression of antioxidant enzymes by sequentially activating the Toll–Akt–Nrf2 pathway. The expression level of *Toll* was upregulated when *T*. *castaneum* was inoculated with a high load of *Enterococcus* sp., partially illustrating the possibility of this regulatory mechanism. Future studies of the molecular mechanisms behind gut microbe-mediated host pesticide resistance with an emphasis on the immune system will inform development of new pesticide targets. For example, the miR-34-5p target gene in the immune pathway of *Spodoptera frugiperda* has been developed as a target of nucleic acid pesticides [43].

## Data Availability

The data presented in this study are available on request from the corresponding author.

## Supplementary Materials

The following supporting information can be downloaded at: https://www.mdpi.com/article/10.3390/insects14100815/s1, Table S1: Primers used for qRT-PCR. Figure S1: Identification of gut bacterial isolates excised from phosphine-resistant *Tribolium castaneum* adults based on 16S rDNA sequence similarity analysis.
