# Supplementary material for: Gut Bacteria Promote Phosphine Susceptibility of Tribolium castaneum by Aggravating Oxidative Stress and Fitness Costs
Source: Insects. 2023 Oct 15;14(10):815. doi: 10.3390/insects14100815 (PMC10607109; doi:10.3390/insects14100815)

Supporting Information

Gut Bacteria Promote Phosphine Susceptibility of *Tribolium castaneum* by Aggravating Oxidative Stress and Fitness Costs

Zhengyan Wang *, Shan Zhang, Zhiyuan Liu, Zhenzhen Chang and Haisheng Hu

School of Food and Strategic Reserves, Henan University of Technology, Zhengzhou 450001, China; 2022920144@stu.haut.edu.cn (S.Z.); 2022920138@stu.haut.edu.cn (Z.L.); 2021920132@stu.haut.edu.cn (Z.C.); 2020920229@stu.haut.edu.cn (H.H.)

* Correspondence: zywang@haut.edu.cn

Table S1 Primers used for qRT-PCR

Primer name	Primer sequence (5´-3´)	Product size (bp)	Amplification efficiency (E%)	Coefficient of determination (R^2)	Usage
16S-27F	AGAGTTTGATCCTGGCTCAG	---	---	---	Bacterial identification
16S-1492R	GGTTACCTTGTTACGACTT				
Tc-*β-actin*-F	TCCATCATGAAGTGCGATGT	228	99.2	1.00	Bacterial quantification
Tc-*β-actin*-R	CCACATCTGTTGGAATGTCG				
16S-F	TCGTTTGAAAATGTCCCT	116	98.2	0.98	Bacterial quantification of
16S-R	GGCGTTGAACTTCCTAAT				*Brevibacterium* sp.
16S-F	AACAATCGAAACCGCATGGT	316	99.6	0.98	Bacterial quantification of
16S-R	TACCGTCAAGGGATGAACAGT				*Enterococcus* sp.
16S-F	TGGAAAGAATTTCGGTCAGGGAT	254	99.4	0.99	Bacterial quantification of
16S-R	ACTTTCGCTTCTTCCCTGCTA				*Microbacterium* sp.
16S-F	TGTGGGTGGGTGGAAAGTTTTT	291	97.4	0.99	Bacterial quantification of
16S-R	CCGGTACTTCTTCTGCAGGTA				*Micrococcus* sp.
16S-F	TGGGTGTTGGAAAGAATTTCGGT	265	98.8	1.00	Bacterial quantification of
16S-R	CGTCACTTTCGCTTCTTCCCTA				*Staphylococcus* sp.
Tc-*Atf2*-F	CCAAACTCCAACTCCAACACG	234	98.7	0.98	Gene expression
Tc-*Atf2*-R	TTCTCACTCGAACTGGGGCT				quantification
Tc-*CAT3*-F	ATCGCAACCCGGAGAACTAC	184	99.0	1.00	Gene expression
Tc-*CAT3*-R	TTGAAGGGACAGTTGACGGG				quantification
Tc-*DUOX*-F	CGATGAGAGTGCCGCTTTTT	250	100.4	0.99	Gene expression
Tc-*DUOX*-R	GCGATGATGTTTTGGACGGT				quantification
Tc-*IMD*-F	CAAGGGCGCTGGATTTCTCA	174	97.8	0.99	Gene expression
Tc-*IMD*-R	CGTCTTGCTGGTTTGTGTCC				quantification
Tc-*PLCβ*-F	AAAACTTGACCCAACGCAGC	190	100.7	0.99	Gene expression
Tc-*PLCβ*-R	TTGGGCTCGTATTGTGCGAT				quantification
Tc-*PRDX6*-F	GGACGAGAAGCGCGAATTG	192	98.5	1.00	Gene expression
Tc-*PRDX6*-R	TGGAGCGAATCAATGACCCT				quantification
Tc-*Rps18*-F	CGAAGAGGTCGAGAAAATCG	235	98.8	0.99	Gene expression
Tc-*Rps18*-R	CGTGGTCTTGGTGTGTTGAC				quantification
Tc-*SOD1a*-F	GCCATCGTGTGCCTCAAATC	238	97.9	0.99	Gene expression
Tc-*SOD1a*-R	TCCCGAGATCACCAACGTG				quantification
Tc-*SOD1b*-F	CCTCCACATTCACCAGTCGG	225	101.1	0.99	Gene expression
Tc-*SOD1b*-R	ACAATCGAACGGCCCACAAT				quantification
Tc-*SOD1c*-F	TGTGTCCTAAACGGCGAAGT	241	97.3	1.00	Gene expression
Tc-*SOD1c*-R	CGATATTCCCCAAATCCCCCA				quantification
Tc-*SOD2*-F	CTCGCTACAGCCCCCTTG	126	98.7	0.99	Gene expression
Tc-*SOD2*-R	GTGGTGTTTGGAGTGGTGGA				quantification
Tc-*Toll*-F	CATGCGTACCGCCAAAAATACA	250	99.3	1.00	Gene expression
Tc-*Toll*- R	ACGCTTCGGGTTAAGTCTACAA				quantification

Figure S1 The neighbour-joining phylogenetic tree of five culturable gut bacteria isolated from adult *Tribolium castaneum*. The tree was constructed from 16S rDNA sequences (1422–1465 bp) using the generalized time-reversible model.

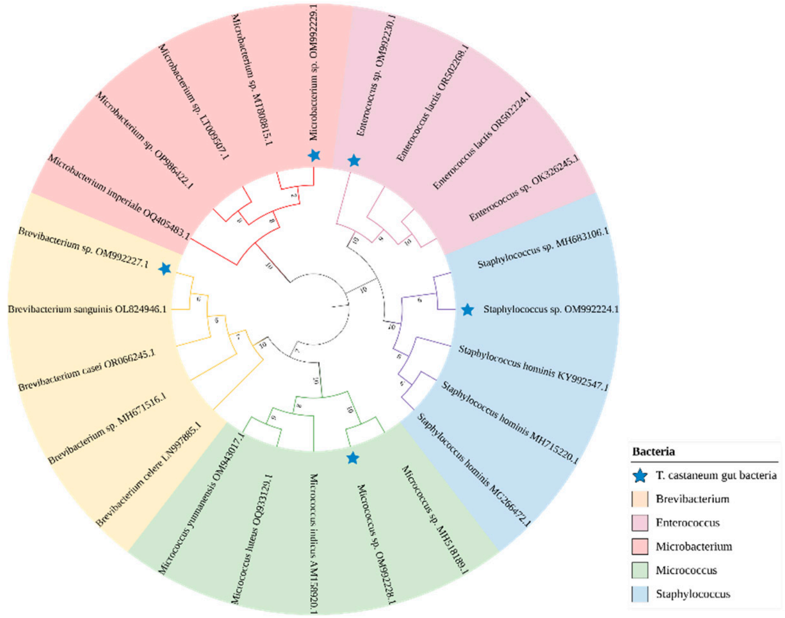

Supplement: Supplementary file 1 [file insects-14-00815-s001.zip › insects-2590402-supplementary.pdf]
